# The Addition of High-Load Resistance Exercises to a High-Intensity Functional Training Program Elicits Further Improvements in Body Composition and Strength: A Randomized Trial

**DOI:** 10.3390/sports10120207

**Published:** 2022-12-13

**Authors:** Georgios Posnakidis, George Aphamis, Christoforos D. Giannaki, Vassilis Mougios, Gregory C. Bogdanis

**Affiliations:** 1 Department of Life and Health Sciences, University of Nicosia, CY-1700 Nicosia, Cyprus; 2 Research Centre for Exercise and Nutrition (RECEN), CY-1700 Nicosia, Cyprus; 3 Laboratory of Evaluation of Human Biological Performance, School of Physical Education and Sport Science, Aristotle University of Thessaloniki, 54124 Thessaloniki, Greece; 4 School of Physical Education and Sport Science, National and Kapodistrian University of Athens, 17237 Athens, Greece

**Keywords:** body fat, fitness, muscle mass, high-intensity exercise

## Abstract

The current study aimed to examine the effects of adding specific high-load resistance exercises to a high-intensity functional training (HIFT) program on healthy adults’ physical fitness and body composition. Twenty recreationally active volunteers (30 ± 4 y, 12 females, 8 males) were randomly assigned to either a HIFT-control (HIFT-C, *n* = 10) or HIFT-power (HIFT-P, *n* = 10) group and trained three times per week for eight weeks. The HIFT-C protocol included four rounds of an 8-exercise circuit (30:15 s work: rest, 2 min rest after the second round). The exercises used were clean-and-press, box jump, TRX chest press, wall ball throws, burpees, repeated 10 m sprints, sumo squat-and-upright row, and abdominal crunches. The HIFT-P-group replaced TRX chest press with bench press and squat-and-upright row with squat, both at an intensity of 80% 1 RM. Before and after the intervention, participants underwent an evaluation of body composition, cardiorespiratory fitness, vertical jump, 1 RM bench press, and the maximum number of abdominal crunches in 1 min. In both groups, cardiorespiratory fitness, squat jump, countermovement jump, bench press 1 RM, and percent body fat improved significantly after the intervention (*p* < 0.050), while a trend towards significant time x group interaction was found for bench press 1 RM (*p* = 0.076), indicating a superiority of HIFT-P over HIFT-C. Muscle mass significantly increased by 3.3% in the HIFT-P group, while abdominal muscle endurance improved by 16.2% in the HIFT-C group (*p* < 0.050). Short-term HIFT resulted in improvements in whole-body cardiorespiratory and neuromuscular fitness and reduction of body fat. The addition of high-load resistance exercises was well tolerated and resulted in increased muscle mass and upper body maximal strength. HIFT-P programs can be suitable for individuals seeking to enhance muscle mass and physical fitness in a short time.

## 1. Introduction

High-intensity functional training (HIFT) is a modern form of exercise which improves several aspects of physical fitness and mental health [1]. HIFT programs combine high-intensity and functional exercises and could improve aerobic and anaerobic exercise performance. The multi-joint (running, rowing, thrusters, etc.) exercises included in a typical HIFT program positively affect several physical fitness and health parameters and body composition [1,2]. It has been proposed that HIFT can be adjusted to any fitness level and that it elicits improvements in body composition [3,4], muscle strength [5], and cardiorespiratory fitness [6,7]. Notably, such programs can be performed safely and without inducing excessive inflammation or muscle damage [7]. Moreover, HIFT can also beneficially affect psychological [8] and health-related quality of life parameters [6]. HIFT programs can be easily utilized to train relatively large groups of individuals and are thus suitable for implementation in sports centers and gyms [7]. Considering these benefits and the time-efficient nature of high-intensity exercise programs, it is not surprising that HIFT has received great popularity.

However, the effect of HIFT on muscle mass and strength-related components of physical fitness remains controversial. Muscle mass and strength are highly associated with sports and exercise performance in athletic populations, while in the general adult population, increases in muscle mass and strength are linked to better quality of life and ability to perform daily activities. Moreover, strength has been associated with all-cause mortality [9].

To our knowledge, the effects of a typical HIFT modality on strength and muscle hypertrophy have not been studied extensively. In a recent study from our group, eight weeks of a group-based HIFT program resulted in a reduction in body fat and improvement in cardiorespiratory fitness in healthy young adults [7]. However, despite the increase in muscle endurance of upper body muscles, there was no increase in muscle mass. Thus, there is a need to modify the typical HIFT modalities to improve such parameters. Indeed, some recent studies examined the effects of various HIFT programs on physical fitness and body composition parameters. For example, 12 weeks of HIFT using low or medium load was equally effective in improving lean body mass and maximal strength, although only the low-load group exhibited reductions in body fat [10]. In addition, participation in CrossFit for six months improved physical fitness parameters, including muscle strength and endurance [5].

It is thought that muscle growth with resistance training can be achieved using 8–12 repetitions per set at 60 to 80% of 1 RM [11,12]. It is also believed that resistance training with a load of 70% 1 RM is sufficient to cause an increase in muscle strength and induce muscle hypertrophy [13]. In addition, training using a wider loading range (e.g., 6–15 RM) can result in significant muscle and strength gains [14]. However, during a typical HIFT program, the participants perform many more than 15 repetitions by using either bodyweight exercises (burpees, push-ups, etc.) or an external load which usually does not exceed 60% of 1 RM (i.e., deadlifts, thrusters). Recent evidence suggests that muscle hypertrophy can be achieved using low loads, provided that sets are preformed to volitional failure, which is not always the case in HIFT [15]. Thus, it is difficult to optimize HIFT in terms of intensity and volume to obtain increases in both muscle mass and strength. Currently, there is a scarcity of studies on the effect of a group-based HIFT program on muscle mass, and there is no specific HIFT program designed to maximize muscle mass gains while being tolerable. Therefore, the purpose of the present study was to examine the effects of replacing one upper-body and one lower-body exercise of a standard HIFT program with similar high-load exercises (80% 1 RM) on muscle mass and strength. We hypothesized that adding the high-load exercises would increase muscle mass and further improve physical fitness parameters in healthy, physically active adults.

## 2. Materials and Methods

### 2.1. Participants

Power analysis using repeated measures within–between interaction analysis of variance (G-Power software, v. 3.1.9.2, Universität Kiel, Kiel, Germany) indicated a minimum sample size of 8 participants per group, based on a power of 0.80, alpha of 0.05, and correlation coefficient of 0.5 between repeated measures. In a relevant study [7], the effect size for similar parameters was between medium and large (partial eta squared values reported were between 0.11 and 0.21). We therefore opted to use a medium effect size in the a priori power analysis for all parameters examined (partial eta squared = 0.137) based on Cohen (1988).

Twenty healthy, physically active adult volunteers participated in this eight-week randomized trial (12 females and 8 males, 30 ± 4 y, mean ± SD throughout). All participants had at least six months of experience in resistance training (including high-load training) and high-intensity training. The inclusion criteria for the study were healthy individuals (males and females from 18 to 45 years of age) without any acute musculoskeletal problem/injury, BMI below 30 kg·m^−2^, and no medication prior or during the study. The exclusion criteria were smoking, musculoskeletal or metabolic disease, and participation in any other form of training additional to the experimental HIFT programs.

A physician screened all participants before the initiation of the study. All participants were healthy and not taking any medication. This study was approved by the National Bioethics Committee of Cyprus (CNBC/2016/56). All participants signed an informed consent form, following a written and verbal explanation of the study’s nature, aim, measurements and possible risks.

### 2.2. Study Design

An independent researcher not involved in data collection or analysis performed a computer-generated randomization taking into account the gender to allocate the subjects equally into the two groups. Participants in both groups performed three HIFT sessions per week. Each session consisted of 4 rounds of 8 exercises (30 s each with 15 s of rest) with a 2 min recovery break after round 2. Each session lasted 36 min (including 5 min of warm-up and 5 min of cool-down). The HIFT-C ((HIFT-control) group; *n* = 10, 6 females) followed a program which included clean-and-press, box jump, TRX chest press, wall ball throws, burpees, repeated 10 m sprints, sumo squat-and-upright row (at 65% 1 RM), and abdominal crunches. The HIFT-P ((HIFT-power) group; *n* = 10, 6 females) performed the same exercises except that two exercises were replaced by similar ones using a load of 80% of 1 RM. In particular, the TRX chest press was replaced by bench press, and the sumo squat-and-upright row was replaced by a squat. The selected exercises used in the HIFT control group were based on a previous study that showed improvements in physical fitness and body composition parameters [7]. Bench press and squat are multi-joint exercises that easily allow additional load and have been shown to improve both maximal strength and muscle mass [13].

Exercise intensity progression was performed by increasing the weights used in each exercise and, thus, the difficulty level in each exercise.

Fitness and body composition assessment was performed on the week before and the week after the training period. The participants were asked to refrain from strenuous exercise for 24 h before the assessment. Participants kept a detailed food and fluid record during the 24 h before the baseline assessment and replicated it before the final assessment, which was performed at least 72 h after the last training session for the participants to have adequately recovered.

Measurements included maximal oxygen uptake (VO_2_ max), peak isokinetic torque of knee extension and flexion, bench press 1 RM, vertical jump, muscle endurance, and body composition assessment. The number of repetitions performed during the 30 s of each exercise during training was also recorded as an index of the participants performance during the workout.

Fitness and body composition assessments were carried out simultaneously in the Human Performance Laboratory of the university. In order to accommodate all participants, there were more than one sport scientist and exercise physiologist administering the tests, and each tester was responsible for a single test. These individuals were blinded as to the group each participant belonged to. Adherence to training was monitored, and no participant missed more than 2 training sessions over the 8-week training period.

### 2.3. Anthropometric and Body Composition Assessment

Anthropometric and body composition assessment was performed in the early morning hours (7–9 a.m.) in the fasted state. Body mass, body fat, and muscle mass were measured with a multi-frequency bioelectrical impedance analyzer (Seca mBCA 515, Seca, Chino, CA, USA). During the assessment, the participants wore light shorts and a T-shirt. Height was measured with a stadiometer to the nearest 0.5 cm (Seca, Chino, CA, USA).

### 2.4. Cardiorespiratory Fitness Assessment

VO_2_ max was measured using a running protocol οn a treadmill (h/p/cosmos pulsar 3p, HP Cosmos, Nussdorf-Traunstein, Germany). The initial speed was set at 8 km·h^−1^ and was increased by 1 km·h^−1^ every minute until exhaustion. A breath-by-breath analyzer (Quark CPET, Cosmed, Rome, Italy) was used to measure oxygen uptake. The ACSM criteria for determining VO_2_ max were used [16]. Heart rate (HR) was continuously monitored via a Polar heart rate monitor (H10 Heart Rate Sensor, Kempele, Finland). The respiratory compensation (RC) point, corresponding to the second ventilatory threshold, was determined based on changes in minute ventilation (VE), VO_2_, and VCO_2_, according to Wasserman [17]

### 2.5. Isokinetic Torque Assessment

Torque of knee extensors and knee flexors was assessed using an isokinetic dynamometer (HumacNorm 770, CSMI Humac Norm, Stoughton, MA, USA). The participants performed 5 maximal single-leg concentric extensions and flexions at an angular velocity of 60°·s^−1^ under verbal encouragement. The highest torque achieved was recorded.

### 2.6. Vertical Jump Performance

Vertical jumps were used as indices of leg explosive power. Two types of jumps, squat jump (SJ) and countermovement jump (CMJ) with free arm swing, were performed using the Opto Jump Next device (Microgate, Bolzano, Italy), which estimates jump height from flight time. The vertical jump assessment was performed following a verbal and practical explanation of the procedure and proper technique by a sport scientist (research group member). During SJ, the participants had to initiate the jump from a squat positing with hands kept firmly at the waist until completion. CMJ was started from an upright position, followed by a quick squat and rebound movement where hands swung freely to provide a further lift. The best of three SJ and three CMJ efforts were recorded. The same person assessed vertical jump at baseline and after the intervention to ensure that the jumping technique was the same during both assessments.

### 2.7. Upper-Body Strength Assessment

Upper body strength was evaluated by the bench press exercise. After a warm-up with light weights, participants performed 10 repetitions at 50% of the estimated 1 RM, followed by 5 repetitions at 75% and 3 at 85%. Then, the weight was increased by 2.5 or 5 kg sequentially to achieve the actual 1 RM. There was a 4 min break between sets. All participants had previous experience in bench press, and an exercise scientist supervised the test. Regarding the two added strength exercises, an assessment of 1 RM test was performed at the end of the first 4 weeks of HIFT, and the weights were re-adjusted to achieve a load of 80% of 1 RM.

### 2.8. Upper-Body Muscle Endurance Assessment

Muscle endurance of the arm and chest muscles was evaluated using the bench press exercise to failure, and muscle endurance of the abdominal muscles was evaluated through the 1 min sit-up test. Both tests were performed after a 10 min warm-up. For bench press, participants were asked to perform as many repetitions as possible at 65% 1 RM with the correct technique. The assessment was terminated when the participant failed to maintain the correct technique or was unable to re-rack the barbell. For the sit-up test, participants were positioned with knees bent at 90°, feet kept firmly on the ground by an assistant, and arms folded behind the neck. They were then asked to perform as many sit-ups as possible within 1 min. A repetition was accepted only if the elbows touched the thighs.

### 2.9. Heart Rate Monitoring

During the first and last sessions of the 8-week training period, HR was monitored using telemetry sensors and software (H7 Polar Team, version 1.2). Peak HR, average HR, and total time during which HR was at or above 90% of maximal heart rate (T-90) were recorded.

### 2.10. Statistical Analyses

The Shapiro–Wilk and Levene’s tests were used to assess normality of distribution and homogeneity of variances, respectively. Two-way (time x group) univariate analysis of variance (ANOVA) with repeated measurements on both factors was used to examine the effect of the training programs on the examined parameters. Were appropriate, further analysis was conducted for exercise performance during round 1 and round 4 of the HIFT session (week (1st vs. 8th) x round (1 and 4)). When a significant interaction was found, Tukey’s post hoc test was used to locate significant differences. Data were analyzed using SPSS for Windows, v.23.0 (IBM, Armonk, NY, USA). Statistical significance was accepted at *p* < 0.05.

## 3. Results

All participants completed the 8-week intervention successfully and without reporting any injuries.

### 3.1. Antrhopometry and Body Composition

Body fat decreased over time (*p* = 0.037, Table 1). There was a significant time x group interaction (*p* = 0.001) with regard to muscle mass. Briefly, muscle mass decreased slightly in the HIFT-C group (*p* = 0.047). There were no significant changes in body mass or BMI over the 8-week training period.

### 3.2. Cardiorespiratory Fitness

A main effect of time was found regarding cardiorespiratory fitness parameters (Table 2). Participants showed significant increases in VO_2_ max, maximum aerobic speed, and speed at the RC point, and a decrease in HR at the RC point after the intervention (all *p* < 0.01). No main effect of group or time x group interaction was observed.

### 3.3. Lower- and Upper-Body Strength

There were no statistically significant findings in any of the isokinetic dynamometer parameters examined (Table 3). In contrast, participants showed increased jumping ability (in terms of both SJ and CMJ) and jumping ability after the intervention. In particular, chest press 1 RM increased by 22% and 11%, respectively (all *p* < 0.001), SJ performance improved by 12% and 8%, respectively (*p* < 0.001) and CMJ performance by 8% and 7%, respectively (*p* < 0.001). A trend towards significant time x group interaction was found in press 1 RM (*p* = 0.076), suggesting a superiority of HIFT-P over HIFT-C.

### 3.4. Upper-Body Muscle Endurance

Training led to significant improvement in number of sit-ups (*p* = 0.003, Table 4). The number of sit-ups was increased by 15% after training only in the HIFT-C group. On the other hand, the number of bench press repetitions at 65% of 1 ME exhibited a marginal increase with training (*p* = 0.089).

### 3.5. Heart Rate Responses during Training Sessions

**Figure 1:**
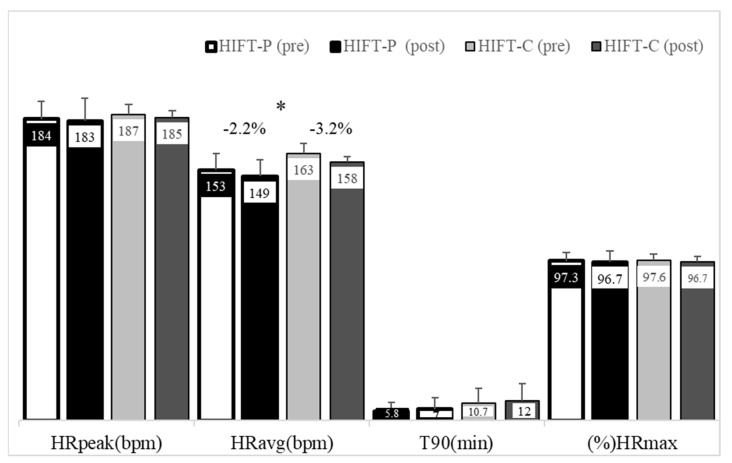
Heart rate responses during the first and last sessions of high-intensity functional training (HIFT). HIFT-P, high-load group; HIFT-C, control group; HRpeak, peak heart rate; HRavg, average heart rate; % HRmax, percentage of maximal heart rate; T90, time during which heart rate was at or above 90% HRmax. * Main effect of time (*p* = 0.003).

During the 8-week training period, there was a decrease in average HR from the first to the last session (*p* = 0.003, Figure 1), with the HIFT-C group experiencing a significantly greater decrease of 3.2% (*p* = 0.021) compared to 2.2% decrease that was observed in the HIFT-P (*p* < 0.050).

### 3.6. Performance during Training Sessions

**Figure 2:**
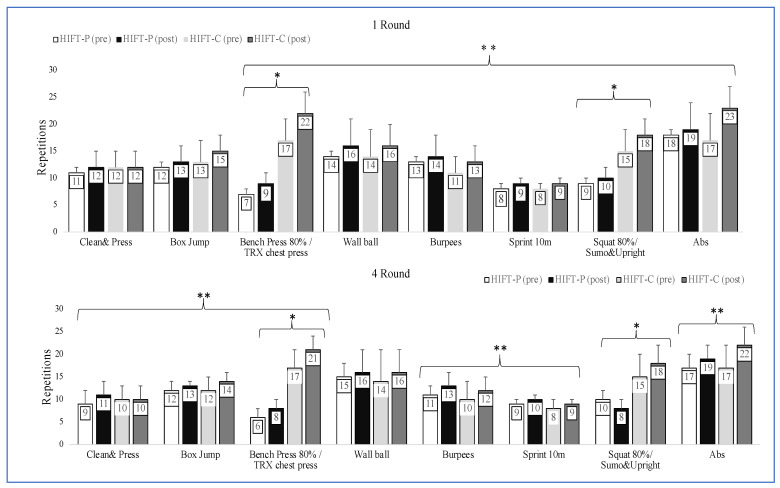
Number of repetitions in each exercise during the first and last sessions of high-intensity functional training (HIFT). HIFT-P, high-load group; HIFT-C, control group. * Main effect of time, ** Main effect of group.

The number of repetitions of the eight exercises included in the training program that were performed by each group at rounds 1 and 4 of the HIFT protocol during the 1st and 8th weeks of training are presented in Figure 2. Regarding performance during round 1, participants showed an increase in box jump, bench press/TRX chest press, wall ball throws, burpees, sprints, squats and abdominal crunches with training (*p* < 0.05). A significant group effect was found in bench press/TRX chest press and squat/sumo squat-and-upright row, while a significant time x group interaction was found in squat/sumo squat-and-upright row (*p* < 0.05). Regarding performance during round 4, participants showed an increase in clean-and-press, box jump, bench press/TRX chest press, burpees, sprints, and abdominal crunches (*p* < 0.05). A significant group effect was found for chest press 80%/TRXchest press, Squat 80%/Sumo and upright while a significant time x group interaction was found in clean-and-press exercise (*p* < 0.05).

## 4. Discussion

This study examined whether the addition of two resistance exercises with high load to a standard HIFT program could elicit further improvements in muscle mass and strength in physically active adults. While both the standard and the modified HIFT programs effectively enhanced various physical fitness parameters, only the modified HIFT program (HIFT-P) resulted in increased muscle mass and upper body maximal strength. Therefore, individuals who use HIFT programs and seek to improve muscle mass and maximal strength should consider modifying their program by including exercises with a higher load.

Recent studies have found that HIFT programs improve aerobic and anaerobic metabolism and related physical fitness components [7,18,19]. This is essential for people who desire to obtain cardiorespiratory and neuromuscular benefits and favorable body composition changes from regular exercise. HIFT programs can reduce body fat [1,8]; therefore, such programs are promoted as an effective approach in terms of weight and fat loss in overweight individuals, as a recent systematic review and network metanalysis showed [20]. In another recent study, only HIFT programs using low-load exercises were able to reduce body fat, as opposed to a HIFT program with moderate-load exercises, which did not [10]. However, the effectiveness of such programs in increasing muscle mass is still debatable. Inducing weight and fat loss with training, parallel to an increase in muscle mass, has always been a great challenge for exercise professionals. Traditional strength and resistance training programs were considered the most appropriate for gaining muscle [13,21]. Similarly, studies using HIFT programs have failed to show improvements in muscle mass, despite a reduction in fat mass and an increase in strength [7]. Indeed, such multi-joint and high-intensity forms of exercise have been recently reported as highly effective in reducing body weight and body fat in overweight and obese populations [20].

A common outcome of high-intensity exercise studies (including HIIT and HIFT studies) is that a reduction in body fat and weight may not be accompanied by an increase in muscle mass [7]. Similar findings have been observed in other HIFT studies, lasting 12–16 weeks [7,22], while other studies did not find a change in body fat or weight after HIFT programs, but they were applied to inactive and overweight/obese individuals [18,23]. In the present study, both HIFT programs were effective in reducing body fat (Table 1). This may be attributed to increased post-exercise fat oxidation, increased growth hormone and epinephrine release, and increased energy expenditure during exercise [24,25,26]. A remarkable finding of the present study was the increased muscle mass only in the HIFT-P group. In a recent study from our group, in which a standard HIFT program was used, body weight and fat decreased, but muscle mass was maintained after the intervention [7]. To our knowledge, this is the first study to report an increase in muscle mass after such an intensive HIFT program. It seems that the inclusion of just two multi-joint exercises with high load can stimulate muscle hypertrophy. According to a recent systematic review and meta-analysis, resistance exercise can induce muscle hypertrophy independently of the training load used in the exercises [15]. However, as stated in the same article, higher loads may offer both muscle hypertrophy and a meaningful improvement in strength. Therefore, the use of one upper- and one lower-body resistance exercise with relatively high load (80% 1 RM) was adequate to induce an increase in muscle mass and strength.

Regarding aerobic fitness, both groups showed improvements, confirming the hypothesis that HIFT can have a beneficial effect on cardiorespiratory fitness, in agreement with recent data [7,27]. A recent systematic review and meta-analysis confirmed the substantial benefits in cardiorespiratory fitness with resistance exercise forms such as HIFT [28], which is considered to be the cornerstone of cardiovascular health, reducing mortality rates [29]. In addition, the HIFT programs studied improved muscle endurance and strength of upper body muscles. According to the literature, muscle endurance can increase due to adaptations within the skeletal muscle such as increased capillary density and mitochondrial content and function [30,31]. Interestingly, muscle endurance of the abdominal muscles (expressed by the number of sit-ups performed in one minute) improved only in the HIFT-C group. This finding can be attributed to the increased demand of performing the exercises of the HIFT-P program, which may have caused fatigue. Notably, an abdominal muscle exercise (abdominal crunches) was performed after the heavy squat in the HIFT-P group, and thus performance could have been affected by fatigue. This is very important in terms of prescribing HIFT as the order of exercises, their nature (i.e., multi-joint) and the relative intensity can affect both acute performance and the subsequent adaptations.

Both training programs were effective in improving maximal strength of upper-body muscles, as expressed by bench press 1 RM. In addition, both programs were effective in improving the performance in the jumping ability tests confirming previous data [27]. However, changes were more remarkable in the HIFT-P group. Thus, adding high-load exercises to HIFT benefited upper-body strength. Exercising using higher loads can result in greater neural adaptations and thus further improve muscle strength and power [32]. Moreover, the inclusion of multi-joint exercises in such programs has been reported to improve 1 RM [21]. Strength is an important contributor to health and quality of life across the lifespan. Adequate strength levels are essential in everyday life, as they are strongly associated with the ability to carry out daily living activities, thus enhancing quality of life [33]. In addition, low strength levels are considered a significant risk factor for current and future health problems [34] and are associated with all-cause mortality [9]. It is noteworthy that recent studies suggest starting strength-related exercise training at an early age to prevent muscle loss in later life and lower the risk of developing chronic diseases and pathological conditions [35]. It is noteworthy that fitness benefits were obtained after just 8 weeks of training in the present study, with exercise sessions lasting only 36 min (including warm-up and cool-down). Lack of time is the main barrier to exercise in modern societies [36]. Thus, such programs fit well with people’s busy programs, as they are time efficient and effective, can be performed in various settings, and can be adopted into the daily schedule.

The current study has some strengths and weaknesses we wish to acknowledge. Body composition was assessed by using a multifrequency BIA analyzer, which is more accurate compared to single-frequency BIA analyzers [37,38]. Nevertheless, assessment using gold-standard methods such as dual X-ray absorptiometry could provide more accurate information about body composition. One of the study’s main strengths is that the research design and assessment techniques used are consistent. Overall, this was a well-executed study with strict supervision of the exercise training program.

## 5. Conclusions

In conclusion, the current study showed that adding only two high-load resistance exercises to a HIFT program elicited increases in muscle mass and a further improvement in strength, in addition to the physical fitness and body composition benefits obtained with a standard HIFT program. The new program was safe and may be suitable for persons seeking to increase muscle mass and strength, along with the cardiorespiratory fitness and muscle endurance benefits already associated with HIFT in the literature, in a time-efficient manner.

## Figures and Tables

**Table 1:** Body composition data before and after 8 weeks of high-intensity functional training (HIFT).

Variable	HIFT-P (*n* = 10)	Change %	HIFT-C (*n* = 10)	Change %	Time	Time x Group	Group
**Muscle mass (kg)**							
Before	23.3 ± 5.6		23.6 ± 7.1				
After	23.9 ± 5.3	2.57%	23.4 ± 7.1	−0.9%	**0.047**	**0.001**	0.975
**Body fat (%)**							
Before	23.6 ± 4.4		22.5 ± 6.7				
After	22.3 ± 4.8	−5.5%	21.9 ± 5.6	−2.7%	**0.037**	0.496	0.652
**Body mass (kg)**							
Before	65.8 ± 12.7		64.5 ± 12.8				
After	65.6 ± 12.3	−0.3%	63.7 ± 12.6	−1.2%	0.274	0.395	0.801
**BMI (kg m^−2^)**							
Before	23.5 ± 3.4		23.1 ± 2.7				
After	23.4 ± 3.3	−0.4%	22.8 ± 2.6	−1.3%	0.160	0.305	0.653

HIFT-P, high-load group; HIFT-C, control group; BMI, Body mass index. Boldface indicates significance at *p* < 0.05.

**Table 2:** Cardiorespiratory fitness-related parameters before and after 8 weeks of high-intensity functional training (HIFT).

Variable	HIFT-P (*n* = 10)	Change %	HIFT-C (*n* = 10)	Change %	Time	Time x Group	Group
**VO_2_ max (mL·kg^−1^·min^−1^)**							
Before	47.0 ± 5.8		44.0 ± 9.2				
After	49.1 ± 7.4	4.3%	46.6 ± 9.2	5.9%	**0.003**	0.487	0.450
**Maximum aerobic speed (km·h^−1^)**							
Before	14.4 ± 1.8		13.4 ± 2.8				
After	14.7 ± 1.8	2.1%	14.3 ± 2.6	6.7%	**0.001**	0.140	0.457
**Speed at RC point (km·h^−1^)**							
Before	11.6 ± 1		10.6 ± 2				
After	12.1 ± 1	4.3%	11.2 ± 2	5.7%	**0.003**	0.899	0.164
**HR at RC point (b·min)**							
Before	173 ± 6		175 ± 7				
After	169 ± 6	−2.3%	171 ± 7	−2.3%	**0.004**	1.000	0.400

HIFT-P, high-load group; HIFT-C, control group; VO_2_ max, maximal oxygen uptake; HR, heart rate; RC, respiratory compensation. Boldface indicates significance at *p* < 0.05.

**Table 3:** Jumping ability and upper-body strength before and after 8 weeks of high-intensity functional training (HIFT).

Variables	HIFT-P (*n* = 10)	Change %	HIFT-C (*n* = 10)	Change %	Time	Time x Group	Group
**SJ (cm)**							
Before	30.1 ± 8.3		27.3 ± 7.5				
After	33.8 ± 7.9	12.3%	29.4 ± 7.6	7.7%	**<0.001**	0.192	0.187
**CMJ (cm)**							
Before	35.4 ± 8.9		32 ± 9.3				
After	38.2 ± 7.8	7.9%	34.1 ± 9.7	6.6%	**<0.001**	0.337	0.236
**Bench press 1 RM (kg)**							
Before	52 ± 18		46.3 ±24.2				
After	62 ±20	19.2%	51.1 ±24.4	10.4%	**<0.001**	0.076	0.372
**Peak torque, right leg extension, 60°·s^−1^ (Nm)**							
Before	170.6 ± 44.6		156.1 ± 66.4				
After	166 ± 49.4	−2.7%	155 ± 64	−0.7%	0.467	0.694	0.603
**Peak torque, left leg extension, 60°·s^−1^ (Nm)**							
Before	173.7 ± 47.6		154.2 ± 55.3				
After	170.8 ± 57.4	−1.7%	153.9 ± 58.2	−0.2%	0.636	0.825	0.471

HIFT-P, high-load group; HIFT-C, control group; SJ, squat jump; CM, countermovement jump. Boldface indicates significance at *p* < 0.05.

**Table 4:** Muscle endurance of upper muscles before and after 8 weeks of high-intensity functional training (HIFT).

Variables	HIFT-P (*n* = 10)	Change %	HIFT-C (*n* = 10)	Change %	Time	Time x Group	Group
**Bench press, 65% 1 RM (reps)**							
Before	21 ± 5		21 ± 5				
After	22 ± 6	4.8%	23 ± 3	9.5%	0.089	0.289	0.790
**Sit-ups in 1 min (reps)**							
Before	42 ± 7		40 ± 10				
After	44 ± 7	4.8%	46 ± 9	15%	**0.003**	**0.023**	0.956

HIFT-P, high load group; HIFT-C, control group; Bold indicates significance at *p* < 0.05.

## Data Availability

Not applicable.
